# The mediation and interaction of depressive symptoms in activities of daily living and active aging in rural elderly: A cross-sectional survey

**DOI:** 10.3389/fpubh.2022.942311

**Published:** 2022-09-14

**Authors:** Xuelian Fu, Yinli Su, Chunyan Zeng, Liqiong Liu, Yang Guo, Yuanyuan Wu

**Affiliations:** ^1^School of Nursing, Xiangtan Medicine and Health Vocational College, Xiangtan, China; ^2^Nursing Department, Children's Hospital Affiliated to Zhengzhou University, Zhengzhou, China

**Keywords:** active aging, activities of daily living, depressive symptoms, rural health, aged

## Abstract

**Background:**

Compared with urban areas, old adults in rural areas have limited access to medical and health resources in China. Active of daily living ability (ADL) decline and depressive symptoms are common in rural older adults. In particular, the depressive symptoms of the elderly in rural areas are often ignored. Thus, it is difficult to realize high-level active aging at the individual level. In order to explore the effects of ADL and depressive symptoms on the active aging of rural elderly, we conducted a survey and analyzed the mediation and interaction effects of depressive symptoms of ADL on active aging.

**Methods:**

From July to November 2019, a cross-sectional study of 945 elderly rural individuals was conducted in three townships in Xiangtan County, China. Active aging, ADL, and depressive symptoms were assessed using the positive aging questionnaire (PAEQ), ADL scale, and depression in old age scale (DIA-S), respectively. PROCESS macro program model 4 and logistic regression were used to explore the mediation and interaction between ADL and depressive symptoms on active aging.

**Results:**

The proportions of rural elderly with an active aging level were 23.5% (well above average), 50.9% (above average), 24.1% (below average), 1.5% (well below average), respectively. The rates of ADL decline and depressive symptoms were 44.7 and 19.7%, respectively. Mediated effect analysis showed that the relationship between ADL and active aging could be partly mediated by depressive symptoms (ab = −0.2382, boot SE = 0.0437), and the 95% confidence interval was [−0.3311, −0.1584]. The mediating effect proportion of the total effect was 30.7%. Logistic regression showed that ADL and depressive symptoms have an interactive additive effect on active aging. The relative excess risk of interaction (RERI), the attributable proportion due to interaction (API), and the synergy index (SI) scores were 13.109, 0.621, and 2.871, respectively. Older adults with ADL decline and depressive symptoms had higher (OR = 21.115) odds of well-below-average active aging compared with older adults with ADL decline (OR = 3.258) or only depressive symptoms (OR = 5.749).

**Conclusion:**

The findings suggest that the association between ADL and active aging is persistent and partly mediated by depressive symptoms, and comorbid depressive symptoms and ADL decline have an additive effect on active aging. Maintaining independence is an important factor for realizing active aging. However, for the rural elderly with ADL decline and low-level active aging, we can promote the realization of high-level active aging at the individual level through the prevention and treatment of depressive symptoms based on multidisciplinary care.

## Introduction

By 2050, one in six people in the world will be over the age of 65 (16%) (1). This highlights the urgent need to develop novel measures means to tackle. Promoting active aging is a proven policy for an aging society (2). Indeed, many regions and countries have adopted active aging as a national policy (3, 4). China has the largest elderly population in the world. In November 2019, China released the *National Medium- and Long-Term Plan for Actively Responding to Population Aging*, which is a strategic, comprehensive document guiding China's active response to population aging in the middle of this century (5).

While various active aging policies are being promoted, the active aging of individuals cannot be ignored. The realization of active aging at the national level is inseparable from the high-level active aging of individuals. On an individual level, active aging is defined as “the striving for elements of well beings through activities relating to a person's goals, functional capacities and opportunities” (6, 7). It is a good quality of life state in which older adults can fully utilize their capacity for physical, mental, and social participation to satisfy their own needs, even those with impaired function (8). Based on this concept, more elderly people would become social value creators rather than a social burden.

Due to poor socioeconomic conditions and insufficient medical care (9), the level of active aging among the rural elderly is lower than that in older urban adults (77.00 ± 30.00 vs. 102.40 ± 19.00, respectively) (10, 11). In order to promote high-level active aging, many researchers have analyzed the factors that influence active aging. A systematic review indicated that the main influencing factors of active aging include health status, psychological factors, marital status, education level, economic status, social support, and living conditions (12). Among these, the physical and psychological conditions of the elderly are the most critical factors (13–15) for rural older adults.

Activities of daily living (ADL) are a common predictor of physical function in the elderly and can reflect the health status of elderly individuals. ADL is often referred to as basic or physical ADL, comprising the basic actions that involve caring for one's self and body, including personal care, mobility, and eating etc. (16). ADL decline is often accompanied by disease and aging (17, 18). About 11–20% of rural older adults experience ADL decline (19, 20) in different countries.

A systematic review proposed that ADL decline could hinder the realization of active aging (12). However, some studies have found that the relationship between ADL decline and active aging is not so closely in advanced ages (15). Therefore, further exploration is needed to determine whether ADL decline is an important factor for achieving active aging among the rural elderly. Although it is not entirely clear how ADL decline affects the realization of high-level active aging at the individual level, there might be a third factor affecting the relationship between ADL decline and active aging, such as pain, body mass index (BMI) (19), social support, or depression (12, 21).

Depression is a global public health problem and one of the most common illnesses among older adults (22). The 2015 China Health and Retirement Longitudinal study reported that the prevalence of depressive symptoms in rural older adults is higher (40.7 vs. 25.2%) than that in urban older adults (23). Researchers have also concluded that depressive symptoms reduce the active aging level in elderly populations (24).

Depression and ADL decline are common and often occur together in the elderly (25). Many studies have confirmed that ADL decline caused by disease often leads to depression in older adults (26, 27). Research on old adults in Pakistan suggested that ADL decline might increase symptoms of depression' rate (28). Moreover, a study showed that lower level of depression can accurately predict high-level active aging (29).

Older adults suffer from ADL decline, resulting in difficulties in performing daily activities, feelings of pain, anxiety, and even desperation (30). These individuals may then seek more help from others in their lives, causing them to feel guilty and useless, which seriously affects their mental health. ADL decline can also lead to psychological distress, such as depression. The combination of ADL decline and depressive symptoms has been associated with higher economic costs than either condition separately (31).

According to the literature analysis described above, ADL decline not only affects active aging but also leads to symptoms of depression in the elderly, and depression plays an important role in low-level active aging.

**Figure 1:**
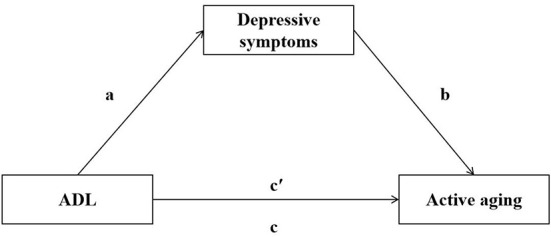
Hypothesis of mediating role of depressive symptoms in ADL and active aging. a, b, c', c represent path coefficients.

Accordingly, we hypothesize that depressive symptoms mediate the relationship between ADL decline and active aging (Figure 1). Further, as ADL decline and depressive symptoms are interrelated, the greater the decline in ADL, the higher the risk of depressive symptoms.

Based on the gaps in the literature and considering advanced hypotheses, this study explores the correlation between ADL decline and active aging and assesses depressive symptoms as an intermediary of this interaction. The results can provide insights on care for the elderly with ADL decline to promote active aging level in rural areas.

## Materials and methods

### Study setting and design

This cross-sectional study was carried out from July to November 2019 in rural areas of Xiangtan County. Xiangtan is a moderately developed city in central China. The population aged 60 and above is 597,872, accounting for 21.93% of the city's population (32). The average life expectancy (78.63 years) of Xiangtan is higher than the national (77.30 years) and provincial (77.10 years) life expectancy.

### Sampling and data collection

First, we divided all 17 administrative villages in Xiangtan County into high, medium, and low groups according to the farmers' per capita annual income level in 2018. Second, we selected a township for each group randomly. Third, we randomly selected three villages in each township. All the random numbers were generated using an on-line random number generation software (https://www.lddgo.net/string/randomnumber). Finally, nine villages were selected. Under the leadership of village staff, we searched for residents who were 60 years or older, did not present with cognitive impairment, and had continuously lived in the village for half a year or more. Then, we explained the purpose of our survey to the elderly participants and the gift they would receive for participation. With their consent, we conducted a face-to-face survey in the villagers' homes. After the survey, we gave a gift (worth about 10 yuan each) to each respondent to express our gratitude.

### Measurement and variables

#### Active aging assessment

Older adults' active aging level was assessed using the positive aging questionnaire (PAEQ) (7). The PAEQ is an individual-level active aging instrument for older Chinese adults. The questionnaire has been widely used and extensively validated in China (33, 34). The measure consists of 21 items and four subscales, including physical vitality, life satisfaction, family support, and active participation. Answers are given on a five-point scale ranging from 1 (no, I totally disagree) to 5 (yes, I totally agree). The total score is the sum of the scores of all items and ranges from 21 to 105 points. The total score is categorized into four levels: well below average (21–42), below average (43–63), above average (64–84), and well above average (85–105). The content validity of the questionnaire was evaluated through a comparison with the literature on the topic and consultation with an expert group. The content validity index (CVI) coefficient of the questionnaire was 1. The credibility and validity of this tool have been established, and it has been used to measure the level of active aging in Chinese elderly adults (33, 34).

#### Activities of daily living assessment

ADL was measured using the ADL scale of Lawton and Brody (35). The scale includes 14 items: walking, eating, dressing, grooming, bathing, and going to the toilet, using transportation, cooking, housework, taking medicine, laundry, shopping, using the phone, and controlling family income. Responses are scored using a four-point Likert-type scale ranging from 1 (“can do it myself”) to 4 (“cannot do it at all”). Older adults were classified as having an ADL decline if they reported limitation in any of the 14 activities. A total score of 14 indicates independence (no ADL decline), whereas a score >14 indicates ADL decline. The Cronbach's alpha coefficient of the ADL scale in this study was 0.918.

#### Depressive symptom assessment

The symptoms of depression in old age were measured by the depression in old age scale (DIA-S). It was designed for seniors by Heidenblut and Zank (36) and has been used in Germany and Iran (37, 38). The scale consists of 10 items, answered in a true–false format. Each item is rated 0 or 1. A score of 3 or more indicates depressive symptoms. The Chinese version was translated by Yang and Guo (39). The total Cronbach's alpha of the Chinese DIA-S was 0.829. In this study, the internal consistency coefficient of the scale was 0.787.

#### Covariates

We collected demographic data, including gender (male or female), age (60–69 or ≥70 years), education years (≤6 or >6 years), marital status (have partner or not), participation in health insurance (participates or not), subjective economic status (poor or good), and chronic disease (have or not).

### Statistical analysis

SPSS 24.0 (IBM Corp.) was used to analyze the data. The participants' characteristics were determined using percentages for description. Based on active aging levels, a Chi-square test was used for comparison of characteristics and ADL and depressive symptoms. We used the PROCESS macro program (40) model 4 (mediating effect) to test the mediating effect of depressive symptoms, using gender, age, education, marital status, participation in health insurance, subjective economic status, and chronic disease as the control variables. In the statistical process, we selected 5,000 bootstrap samples and took the robust standard errors and bootstrap confidence intervals of the parameter estimates. If the confidence interval does not include 0, it means that the mediating effect exists.

The additive interaction between ADL and depressive symptoms was explored by calculating three indicators of interaction on an additive scale: the relative excess risk of interaction (RERI), the attributable proportion of interaction (API), and the synergy index (SI) (41). According to the method described by Andersson et al. (42), RERI = 0, API = 0, and SI = 1 show no additive interaction. First, we set up a variable with four categories: (1) joint ADL decline and depressive symptoms, (2) ADL decline only, (3) depressive symptoms only, and (4) no ADL decline and no depressive symptoms. We performed an ordinal logistic regression analysis with the new variable as the independent variable and active aging (1: well above average; 2: above average; 3: below average; 4: well below average) as the dependent variable, with gender, age, education, marital status, participation in health insurance, subjective economic status, and chronic disease as the control variables. The no ADL decline and no depressive symptoms group was used as a reference standard. Based on the estimated values, we calculated the ORs and their 95% confidence intervals (CIs). In addition, we used an Excel sheet (available from https://www.docin.com/p-1701947013.html) to calculate the three indicators (RERI, API, SI) and their 95% CIs.

## Results

### Participant characteristics

Of the 945 elderly individuals who participated in our study, 10 (1.1%) did not complete the entire questionnaire and were excluded from data analyses. Data on 935 rural older adults were included in the analyses. Table 1 shows the characteristics of rural older adults with different levels of active aging. Of the participants, 477 (51.0%) were males, and 498 (53.3%) were ≥70 years of age. Most of them completed 6 years or less of education and suffered from one or more chronic diseases. There were statistically significant differences in demographics and health characteristics, except for age, and participation in health insurance.

**Table 1:** Distribution of characteristics by active aging status (*n*, %).

**Variables**	**Total (*n* = 935)**	**Active aging level**				**Pearson χ^2^/fisher's exact test**	***P*-value**
		**Well above average (*n* = 220, 23.5%)**	**Above average (*n* = 476, 50.9%)**	**Below average (*n* = 225, 24.1%)**	**Well below average (*n* = 14, 1.5%)**		
Gender (male)	477 (51.0%)	130 (59.1%)	235 (49.4%)	104 (46.2%)	8 (57.1%)	8.536	0.036^*^
Age (≥70)	498 (53.3%)	114 (51.8%)	246 (51.7%)	131 (58.2%)	7 (50.0%)	2.946	0.400
Education (≤6 years)	718 (76.8%)	141 (64.1%)	374 (78.6%)	191 (84.9%)	12 (85.7%)	28.243	<0.001^***^
Marital status (no partner)	273 (29.2%)	48 (21.8%)	126 (26.5%)	92 (40.9%)	7 (50.0%)	24.640	<0.001^***^
Subjective economic status (poor)	318 (34.0%)	36 (16.4%)	151 (31.7%)	119 (52.9%)	12 (85.7%)	84.422	<0.001^***^
Chronic disease (have one or more)	741 (79.3%)	132 (60.0%)	382 (80.3%)	214 (95.1%)	13 (92.9%)	88.967	<0.001^***^
Participation in health insurance (no participation)	201 (21.5%)	46 (20.9%)	107 (22.5%)	45 (20.0%)	3 (21.4%)	0.642	0.897
ADL decline (ADL score>14, dependent)	418 (44.7%)	48 (21.8%)	207 (43.5%)	151 (67.1%)	12 (85.7%)	102.123	<0.001^***^
Depressive symptoms (DIA-S score ≥3, suffer from depression symptom)	184 (19.7%)	10 (4.5%)	62 (13.0%)	100 (44.4%)	12 (85.7%)	155.773	<0.001^***^

ADL, activities of daily living; DIA-S, the depression in old age scale.

* P < 0.05;

*** P < 0.001.

### The active aging level, ADL, and depressive symptoms of participants

A total of 23.5% of the rural elderly participants were at a well-above-average level of active aging (*n* = 220). The prevalence of low-level active aging (including below-average and well-below-average level) was 25.6%.

Of the 935 participants, 418 (44.7%) reported ADL decline, and 184 (19.7%) had depressive symptoms. In the below-average level of active aging group, about 67.1% of elderly adults had ADL decline, and 44.4% suffered from depressive symptoms. In the well-below-average level of active aging group, the proportion was higher, with 85.7% of the participants suffering from ADL decline and depressive symptoms.

Older adults with low-level active aging reported a higher prevalence of ADL decline (*P* < 0.001) and depressive symptoms (*P* < 0.001). Moreover, the participants with low-level active aging were more likely to have fewer years of education, to have no partner, to have a poor subjective economic status, and to suffer from chronic disease (Table 1).

### Analysis and test of the mediating effect of depressive symptoms in the elderly in rural areas

**Figure 2:**
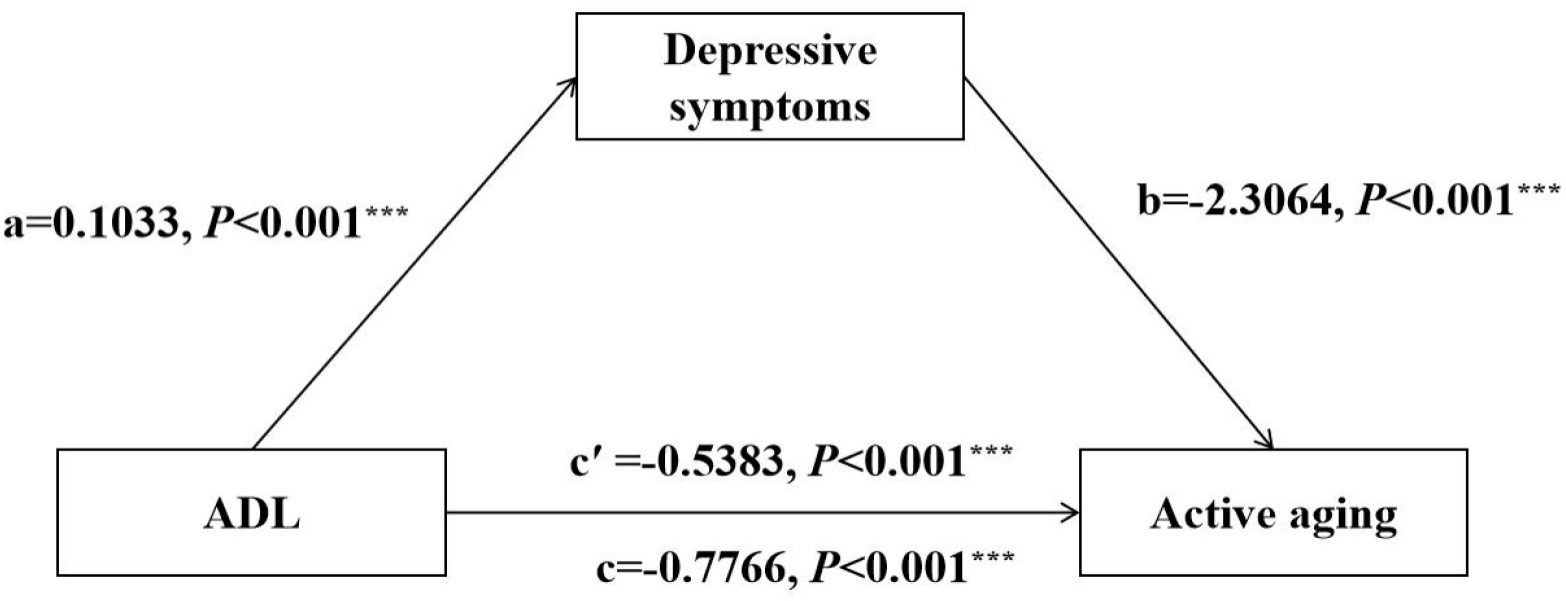
Mediation effect of depressive symptoms in the relationship between ADL and active aging. ADL, activities of daily living. The *** symbol indicates the value of *P* < 0.001.

We verified the mediating effect of depressive symptoms on ADL and active aging using the PROCESS program in SPSS. The mediating effect analysis of depressive symptoms showed that, after controlling the covariates, ADL significantly and positively predicted the depressive symptoms of rural elderly participants (*a* = 0.1033, standard error [SE] = 0.0122, *P* < 0.001), and the depressive symptoms of rural elderly participants significantly and negatively predicted active aging (*b* = −2.3064, SE = 0.1714, *P* < 0.001). The direct effect of active aging on ADL was significant (*c*′ = −0.5383, SE = 0.0659, *P* < 0.001) (Figure 2). The bootstrap results showed a statistically significant mediating effect of depressive symptoms on ADL and active aging (ab = −0.2382, boot SE = 0.0437), and the 95% CI was [−0.3311, −0.1584] (Table 2). The mediating effect proportion of the total effect was 30.7%.

**Table 2:** Mediating effect of depressive symptoms between ADL and active aging.

**Model pathways**	**Effect**	**Boot SE**	**95% CI**
Total effect: ADL → active aging	−0.7766	0.0694	(−0.9127, −0.6404)
Direct effect: ADL → active aging	−0.5383	0.0659	(−0.6677, −0.4090)
Indirect effect: ADL → depressive symptoms → active aging	−0.2382	0.0437	(−0.3311, −0.1584)

ADL, activities of daily living; SE, standard error; CI, confidence interval. An empirical 95% confidence interval does not overlap with zero.

### Interaction of depressive symptoms and ADL decline on active aging

**Figure 3:**
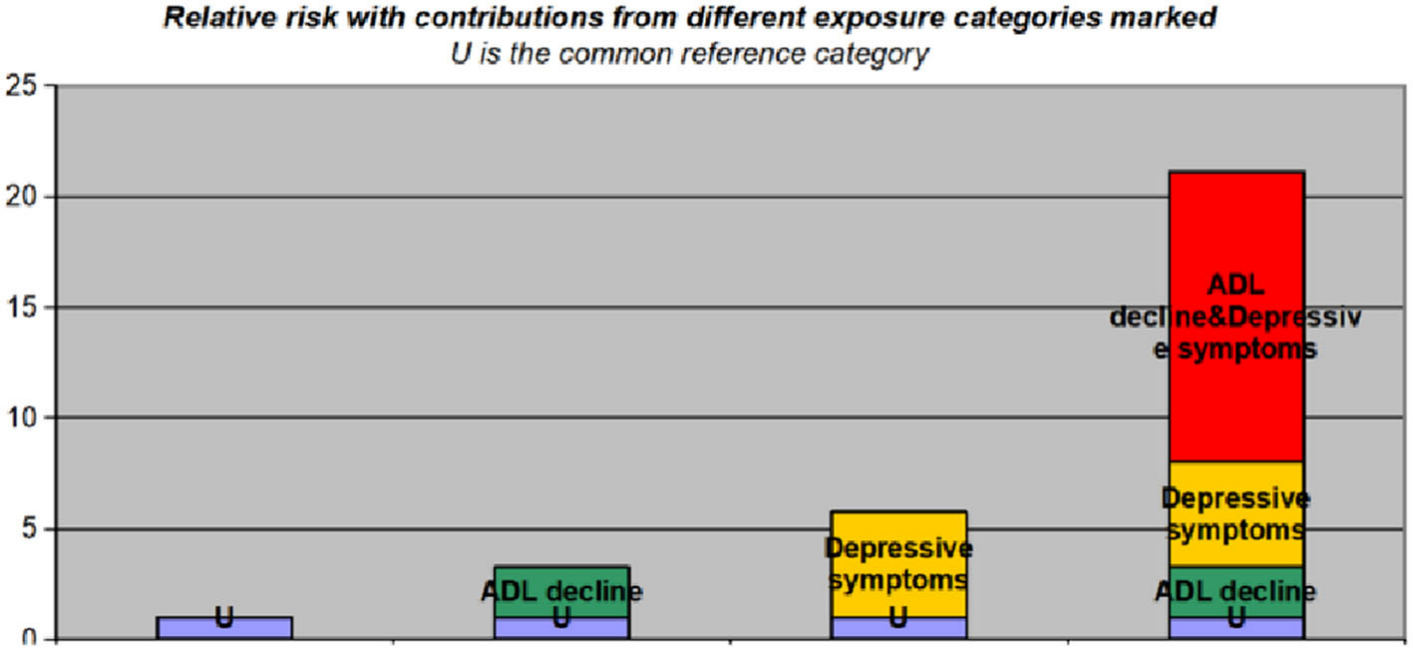
The odds ratio of well-below-average level active aging with contributions from different exposure categories marked (ADL decline and depressive symptoms). U is the reference category (no ADL decline and no depressive symptoms). ADL, activities of daily living.

Table 3 shows the interaction of depressive symptoms and ADL decline on active aging based on an ordinal logistic regression model. The ORs for well-below-average level active aging were 3.258 and 5.749, respectively, for participants with only ADL decline or depressive symptoms. However, for participants with both ADL decline and depressive symptoms, the well-below-average level active aging regulated OR rose substantially to 21.115. The RERI was 13.109 (95% CI: 2.955–23.262), the API was 0.621 (95% CI: 0.414–0.827), and the SI was 2.871 (95% CI: 1.590–5.184). The findings showed that ADL and depressive symptoms had an additive effect on active aging. The two factors worked together to increase the risk of well-below-average level active aging. Figure 3 shows the well-below-average level active aging ORs, annotating the contributions of different exposure categories (ADL decline and depressive symptoms).

**Table 3:** Interaction effect of depressive symptoms with ADL decline on active aging with an ordinal logistic regression.

**Exposure**	**Reference**	**Estimate (95%CI)**	**OR (95%CI)**	***P*-value**
Only ADL decline	No ADL decline and no depressive symptoms	1.181 (0.866–1.495)	3.258 (2.375–4.468)	<0.001^***^
Depressive symptoms only		1.749 (1.250–2.248)	5.749 (3.488–9.475)	<0.001^***^
ADL decline and depressive symptoms		3.050 (2.543–3.558)	21.115 (12.714–35.070)	<0.001^***^
Gender (male)	Female	0.045 (−0.222–0.311)	1.046 (0.801–1.365)	0.741
Age (≥70)	<70 years	−0.316 (−0.590 to −0.042)	0.729 (0.554–0.959)	0.024^*^
Education (≤6 years)	>6 years	0.560 (0.239–0.882)	1.751 (1.27–2.416)	0.001^**^
Marital status (no partner)	Have partner	0.699 (0.405–0.993)	2.012 (1.499–2.699)	<0.001^***^
Subjective economic status (poor)	Good	1.096 (0.809–1.384)	2.992 (2.246–3.991)	<0.001^***^
Chronic disease (have one or more)	No chronic diseases	1.269 (0.929–1.609)	3.557 (2.532–4.998)	<0.001^***^
Participation in health insurance (no participate)	Participation in health insurance	−0.057 (−0.374–0.260)	0.945 (0.688–1.297)	0.724
Model fit	−2 Log likelihood = 776.551 (*P* < 0.001^***^)			
	Pseudo *R*^2^ = 0.388			

The dependent variable is active aging: 1, well above average; 2, above average; 3, below average; 4, well below average (reference).

ADL, activities of daily living; OR, odds ratio; CI, confidence interval.

* P < 0.05,

** *P* < 0.01,

*** *P* < 0.001.

## Discussion

This study reports the active aging of the rural elderly in Xiangtan County. ADL decline and depressive symptoms are important factors affecting the active aging of rural elderly. Depressive symptoms plays a mediate and an interactive additive effect in the process of achieving a high level of active aging of rural elderly with ADL decline. Given ADL decline and depressive symptoms interact with each other, there is an urgent need to integrate the treatment and management of physical function and mental health problems of the elderly into the elderly health care services in rural area.

Physical function and mental health are crucial for achieving a high level of active aging. In our study, in the well-above-average level of active aging group indicts the participants were more independent and had fewer depressive symptoms. It is consistent with the results found in an annual review report (43).

Further, ADL was significantly associated with active aging, which supports the findings of previous studies (24, 44, 45). ADL decline has been linked to lower physical activity, lower life satisfaction, and lower social participation, which are essential components of a low-level active aging state.

Elderly individuals with depressive symptoms are likely to be sedentary and less willing to talk with others (29). In the long run, their physical function and social participation will gradually decline, and they may even develop suicidal tendencies (46). A high level of active aging is also difficult to achieve.

We also found that depressive symptoms are a mediator between ADL and active aging. This helps to explain how ADL decline can lead to low-level active aging. The results of the mediation effect analysis indicated that depressive symptoms partially mediated the link between ADL decline and active aging, attenuating the association by 30.7%. These results are similar to those of a Korean study on ADL decline and quality of life in cancer patients, in which depression played a fully mediating role (47). Some studies have shown that depression is often the result of ADL decline (48), whereas depression is also a risk factor for ADL decline (26). Previous studies have reported that a decline in ADL is associated with depressive symptoms in patients with type 2 diabetes and cardiovascular disease (49). Structural brain abnormalities in depression overlap with ADL controllers in the adult brain (50); thus, ADL decline could have positive effects on depressive symptoms. Regarding psychological and social aspects, ADL decline leads to disability and unproductivity, so people who are in ADL decline will have difficulty performing social and physiological functions, causing people to isolate themselves and become depressed later in life (51, 52). The correlation between ADL and depression is fairly common (53).

In addition, we found that ADL decline and depressive symptoms significantly interacted with active aging. Elderly participants with ADL decline and depressive symptoms were more likely to experience low-level active aging than those with both conditions alone. Depressive symptoms may contribute to ADL decline through lifestyle changes and increased family burden (21). When old adults suffer from ADL decline and depression together, they may have a more severe drop in ADL, which can worsen depression. In rural primary healthcare, many medical institutions usually focus on physical symptoms first, and depressive symptoms are often undetected and easily ignored as a natural phenomenon of aging (54). Due to the shortage of grassroots psychological workers, depression is more difficult to identify in rural elderly individuals. Thus, underdiagnosed and undertreated depressive symptoms may arise in the context of both ADL decline and depression (21). In this situation, it is challenging to support individual active aging.

## Recommendations

This study has several implications for active aging interventions targeting elderly individuals with ADL decline. First, effective management and improvement of ADL should be the top priority in accelerating the process of active aging. Due to the lack of health knowledge on elderly individuals in rural areas, ADL decline is often regarded as a normal aging phenomenon, and they rarely go to the hospital for formal treatment (50–52). It is necessary to increase awareness and provide education about physical rehabilitation for the rural elderly population. It is particularly important for primary health workers to promote the evaluation and comprehensive management of ADL of the elderly. ADL decline is not an inevitable phenomenon of aging. As long as there is a proper environment and adequate help, dependence can be changed, reduced, or even prevented through drug and non-drug treatment (53).

In addition to ADL decline, we need to pay attention to the depressive symptoms of elderly individuals. Depression sometimes manifests initially as an unexplained complaint of ADL decline, which may confound the diagnosis. More psychological workers are needed in rural grassroots health service systems. This would aid in the discovery and screening of rural elderly individuals for depressive symptoms and improve the prevention and treatment of depression in this population. When the physical function of the rural elderly has declined, preventing or treating depression in a timely manner can promote high-level active aging. For rural elderly individuals who have depressive symptoms, we can provide continuous and personalized psycho-social interventions, such as physical exercise, skill training, reminiscence, social activities, group support, and multi-component interventions (55). This would help to improve the mental health and quality of life of the elderly and promote active aging.

Above all, when an individual experiences an unexplained decline in ADL, the possibility of depression should be considered.

## Conclusions

This study emphasizes the need to pay attention to depressive symptoms in the process of achieving a high-level active aging of rural elderly. Preventing the occurrence of depression and improving the depressive symptoms efficiently are important goals to improve the level of active aging in older adults with ADL decline.

## Data availability statement

The raw data supporting the conclusions of this article will be made available by the authors, without undue reservation.

## Ethics statement

The studies involving human participants were reviewed and approved by the Ethics Committee of Xiangya Nursing College of Central South University (no. 2019013). The patients/participants provided their written informed consent to participate in this study.

## Author contributions

XF: conceptualization, investigation, data curation, formal analysis, writing—original draft, and writing review and editing. YS: project administration and resources. CZ and LL: investigation, data curation, and formal analysis. YG: writing review and editing and resources. YW: writing review and editing. All authors contributed to the article and approved the submitted version.

## Funding

This research was supported by a project from the Hunan Provincial Health Commission with grant number 20200094.

## Conflict of interest

The authors declare that the research was conducted in the absence of any commercial or financial relationships that could be construed as a potential conflict of interest.

## Publisher's note

All claims expressed in this article are solely those of the authors and do not necessarily represent those of their affiliated organizations, or those of the publisher, the editors and the reviewers. Any product that may be evaluated in this article, or claim that may be made by its manufacturer, is not guaranteed or endorsed by the publisher.
